# Associations between genetic risk variants for kidney diseases and kidney disease etiology

**DOI:** 10.1038/s41598-017-13356-6

**Published:** 2017-10-24

**Authors:** Sebastian Wunnenburger, Ulla T. Schultheiss, Gerd Walz, Birgit Hausknecht, Arif B. Ekici, Florian Kronenberg, Kai-Uwe Eckardt, Anna Köttgen, Matthias Wuttke

**Affiliations:** 1 Institute of Genetic Epidemiology, Medical Center - University of Freiburg, Faculty of Medicine, Freiburg, Germany; 2 Division of Nephrology, University of Freiburg, Faculty of Medicine, Freiburg, Germany; 3 Department of Nephrology and Hypertension, University of Erlangen-Nürnberg, Erlangen, Germany; 4 Institute of Human Genetics, Friedrich-Alexander-Universität Erlangen-Nürnberg (FAU), Erlangen, Germany; 5 Division of Genetic Epidemiology, Department of Medical Genetics, Molecular and Clinical Pharmacology, Medical University of Innsbruck, Innsbruck, Austria

## Abstract

Chronic kidney disease (CKD) is a global health problem with a genetic component. Genome-wide association studies have identified variants associated with specific CKD etiologies, but their genetic overlap has not been well studied. This study examined SNP associations across different CKD etiologies and CKD stages using data from 5,034 CKD patients of the German Chronic Kidney Disease study. In addition to confirming known associations, a systemic lupus erythematosus-associated risk variant at *TNXB* was also associated with CKD attributed to type 1 diabetes (p = 2.5 × 10^−^^7^), a membranous nephropathy-associated variant at *HLA-DQA1* was also associated with CKD attributed to systemic lupus erythematosus (p = 5.9 × 10^−^^6^), and an IgA risk variant at *HLA-DRB1* was associated with both CKD attributed to granulomatosis with polyangiitis (p = 2.0 × 10^−4^) and to type 1 diabetes (p = 4.6 × 10^−11^). Associations were independent of additional risk variants in the respective genetic regions. Evaluation of CKD stage showed a significant association of the *UMOD* risk variant, previously identified in population-based studies for association with kidney function, for advanced (stage ≥G3b) compared to early-stage CKD (≤stage G2). Shared genetic associations across CKD etiologies and stages highlight the role of the immune response in CKD. Association studies with detailed information on CKD etiology can reveal shared genetic risk variants.

## Introduction

The prevalence of chronic kidney disease (CKD) is high with >10% of the adult population affected in many countries^1^. Its genetic architecture is complex and incompletely understood. Genome-wide association studies (GWAS) have helped to gain insight into complex disease genetics^2,3^ by identifying single-nucleotide polymorphisms (SNPs) in >70 independent risk loci associated with the estimated glomerular filtration rate (eGFR), CKD disease risk and microalbuminuria (MA)^4–6^ as well as specific kidney diseases such as IgA^7,8^ or membranous nephropathy^9^ in case control studies. Because many of these specific kidney diseases are individually rare, only very few studies have collected sufficient numbers of patients with CKD attributed to various of these specific etiologies using one study design and protocol. Consequently, genetic risk variants identified in association with a specific etiology of CKD have so far not been examined for their association with CKD attributed to other etiologies. Capitalizing on data from the large German Chronic Kidney Disease (GCKD) study, we therefore aimed to systematically examine whether risk loci discovered for specific etiologies of CKD, especially for autoimmune conditions^10^, are associated with other CKD etiologies as well. Additionally, we aimed to examine whether risk loci discovered in the general population are also associated with advanced stages of CKD and with CKD in patients for whom the leading cause of disease was hypertension or diabetes, the most common causes of CKD.

## Subjects and Methods

The GCKD study^11,12^ is an ongoing prospective observational study of 5,217 patients under nephrological care, followed for up to 10 years. At enrolment, all patients had CKD defined as an estimated glomerular filtration rate (eGFR) of 30–60 mL/min/1.73 m^2^ or either a urinary albumin-to-creatinine ratio (UACR) >300 mg/g or a protein-to-creatinine ratio >500 mg/g when eGFR was >60 mL/min/1.73 m^2^. The GCKD study was approved by local ethics committees and registered in the national registry of clinical studies (DRKS 0003971). All methods were carried out in accordance with relevant guidelines and regulations. Written informed consent was obtained from all subjects. Case groups for all analyses of specific CKD etiologies were derived based on the leading cause of CKD, which was determined by using standardized case report forms by the treating nephrologist. Serum creatinine was measured using an IDMS traceable gold-standard method. eGFR was calculated using the CKDepi equation^13^. Regardless of CKD etiology, two case groups of “advanced CKD” status were defined and included all patients with eGFR <45 ml/min/1.73 m² (stage G3b+ , n = 2245) or UACR ≥ 300 mg/g (stage A3, n = 1385), respectively. As controls, 1,655 GCKD patients for whom CKD etiology was assigned to a cause that could reasonably be assumed to differ from the case groups (nephrosclerosis, infections, tumor nephrectomies, interstitial nephritis and vascular diseases) were used as the control group for the etiology-specific analyses. For the examination of stage G3b+ and A3 CKD, GCKD patients with eGFR ≥ 45 ml/min/1.73 m² (n = 1006) and UACR < 30 mg/g (n = 2117) were selected as control groups, respectively.

In the GCKD study, 5,123 participants were genotyped for 2,612,357 markers at the Helmholtz Center Munich using the Illumina Infinium Omni 2.5 Exome-8 microarray (Illumina, GenomeStudio, Genotyping Module Version 1.9.4). Data cleaning was performed according to standard protocols^14,15^. Plink v1.90, R programming language and custom shell scripts were used during cleaning. Individual-level data were filtered for missingness (<3%) and mean heterozygosity (>2 SD). Sex checks were performed. These checks resulted in 57 individuals being filtered. Identity-by-descent (IBD) allele sharing measure calculations were used to check for unrecognized and cryptic relatedness. Across 1.28 × 10^7^ evaluated pairs, we detected 11 with the proportion of alleles shared IBD of >0.1875 (between second and third degree relatives). For these pairs, we removed one individual prior to data analysis. Principal component analyses (PCA) using the software Eigenstrat SmartPCA^16^ were conducted to examine and account for population stratification. Outliers were removed in terms of genetic ancestry (automatic outlier detection, taking into account 10 PCs, deviation >8 SD). SNPs were filtered for callrate (>96%), minor allele frequency (>1%) and for deviation from the Hardy-Weinberg equilibrium (p > 1 × 10^−5^). Genotype imputation using the 1000 Genomes Phase 3 ALL reference panel^17^ was conducted according to standard protocols^14,15^. Imputation quality was assessed using the Impute2 “info” measure and was >0.8 for all SNPs. The final dataset after QC contained 5,034 individuals with genotypes for 2,337,794 SNPs.

A literature search was performed to assemble a table of all previously reported SNPs that were associated with either the kidney function measures eGFR and UACR, or with general or etiology-specific CKD risk in populations of European ancestry at genome-wide significance (p < 5 × 10^−8^) and with evidence for replication^3^.

Descriptive statistics were derived as percentages and frequency distributions for all categorical variables and mean and standard deviation or median and interquartile ranges for continuous variables depending on their distribution.

Thresholds of statistical significance were defined for each analysis using a Bonferroni correction to account for multiple testing and set to p < 0.05/n for two-sided hypothesis testing, with n as the number of tested SNPs multiplied by the number of phenotypes. For the analyses comparing etiologies, we used α = 0.05/(38*5) = 2.6 × 10^−4^, for the analyses comparing hypertensive and diabetic nephropathy, we used α = 0.05/(55*2) = 4.5 × 10^−4^. Power calculations have been performed using the software package Quanto^18^.

Multivariable adjusted logistic regression analyses was used to evaluate the association between CKD etiology and genotype dosage assuming an additive genetic model, and accounted for sex and age as covariates. Conditional analyses accounted for further SNPs in addition. We did not adjust the logistic models for principal components as the genetic ancestry of the study population was very homogenous because of the study design and data cleaning. Sensitivity analyses adjusting for prinicipal components were carried out to verify that results were robust. STATA 13.0 (StataCorp., College Station, TX) was used to perform all analyses.

## Results

Association studies were performed using different case definitions as the outcome and genotype data from 38 + 55 = 93 selected SNPs^3^ as the exposure (Subjects and Methods). All GCKD patients were of European ancestry, 60% of them were male. Mean age at study entrance was 60 ± 12 years, and mean eGFR was 49.5 ± 18.1 ml/min/1.73 m² (Table 1).

**Table 1:** Demographic data and baseline characteristics in the GCKD cohort.

Characteristics		N with available data
Demographic and anthropometric data		
Male	60.1 (3027)	5034
Age, years	60.1 (12.0)	5034
BMI, kg/m²	29.8 (6.0)	4982
Blood pressure		
SBP, mm Hg	139.5 (20.4)	5002
DBP, mm Hg	79.2 (11.7)	
Kidney function measures		
eGFR, ml/min	49.5 (18.1)	4993
Serum creatinine, mg/dl	1.5 (0.5)	4993
UACR, mg/g – median (IQR)	50.52 (9.53, 385.26)	4950
<30 mg/g	42.8% (2117)	4950
30–299 mg/g	29.2% (1448)	
≥300 mg/g	28.0% (1385)	
Diabetes mellitus		
Type 1 DM	2.1% (103)	5034
Type 2 DM	24.5% (1231)	5034
HbA_1_c, %	6.3% (1.0)	4941
Leading cause of CKD		
MN	2.9% (147)	5034
IgA	7.3% (366)	
SLE	2.5% (128)	
GPA	2.3% (116)	
T1DM	1.8% (91)	
Positive family history	28.2% (1260)	4475

Table summarizes data of all patients included in the analyses (n = 5034). Continuous variables are described in mean (SD), categorical variables in % (n) unless described otherwise. BMI: Body mass index, SBP: systolic blood pressure, DBP: diastolic blood pressure.

We tested 38 known etiology-specific risk loci for association with additional CKD etiologies across the broad spectrum of CKD etiologies available in the GCKD study: IgA nephropathy (n = 366), membranous nephropathy (MN, n = 147), systemic lupus erythematosus (SLE, n = 128), granulomatosis with polyangiitis (GPA, n = 116) and type 1 diabetes mellitus (T1DM, n = 91; Supplementary Table 1). The proportion of patients with a renal biopsy supporting the diagnosis was 85% (641/757) for these case groups except for T1DM (Table 1). The respective case groups were compared to a control group, which consisted of n = 1655 patients for whom CKD etiology was assigned to a cause which could reasonably be assumed to differ from the case groups (see Subject and Methods). Several known associations^7,9,10,19–21^ were replicated: both *HLA-DQA1* and *PLA2R1* were strongly associated with MN (p = 2.4 × 10^−22^ and p = 6.7 × 10^−8^, respectively), *STAT4* was associated with SLE (p = 9.7 × 10^−5^) and *HLA-DPB1* with GPA (p = 1.7 × 10^−11^, Table 2, Supplementary Table 2), supporting an appropriate selection of the control group. These associations had consistent directions with previously reported associations. Only 4 of 38 (11%) previously reported etiology-specific risk loci showed significant associations after applying the Bonferroni threshold, but many of them had been found and reported from meta-analyses assembling a much larger number of cases. For 21 variants previously reported as associated with IgA nephropathy, 15 displayed associations in the same direction (i.e. the same risk allele) in our data, which is significantly more than expected by chance (p-binomial for observing 15 or more direction-consistent associations = 0.021). Of the 21 variants, 6 were nominally significant (p < 0.05). The effect sizes were similar on average (median effect size difference 0%, inter-quartile range −9% to 6%).

**Table 2:** Associations between CKD etiology associated SNPs and other CKD etiologies.

SNP Characteristics				IgA		MN		SLE		T1DM		GPA	
SNP (*Gene*)	Effect allele	Chr. (Position)	Known locus	OR [95% CI]	P-value	OR [95% CI]	P-value	OR [95% CI]	P-value	OR [95% CI]	P-value	OR [95% CI]	P-value
rs4664308 *(PLA2R1)*	G	2 (160917497)	MN	(1.04 [0.87–1.25])	(6.5 ·10^−1^)	***0.45 [0.34–0.60]***	***6.7 ·10*** ^*−8*^	(0.83 [0.61–1.13])	(2.5 ·10^−1^)	(1.36 [1.01–1.84])	(4.4 ·10^−2^)	(1.03 [0.79–1.36])	(8.1 ·10^−1^)
rs7574865 *(STAT4)*	G	2 (191964633)	SLE	(0.90 [0.74–1.11])	(3.4·10^−1^)	(0.96 [0.71–1.28])	(7.6 ·10^−1^)	***0.53 [0.39–0.73]***	***9.7 ·10*** ^*−5*^	(0.85 [0.60–1.19])	(3.4 ·10^−1^)	(1.02 [0.74–1.40])	(9.2 ·10^−1^)
rs1150754 *(TNXB)*	T	6 (32050758)	SLE	(1.07 [0.83–1.40])	(5.9 ·10^−1^)	2.77 [2.06–3.71]	1.9 ·10^−11^	*(1.97 [1.37–2.83])*	*(2.8 ·10* ^*−4*^ *)*	**2.53 [1.78–3.60]**	**2.5 ·10** ^−7^	(0.94 [0.63–1.42])	(7.7 ·10^−1^)
rs660895 *(HLA-DRB1)*	G	6 (32577380)	IgA	*(1.09 [0.86–1.37])*	*(4.7 ·10* ^*−1*^ *)*	(0.60 [0.40–0.89])	(1.2 ·10^−2^)	(1.15 [0.77–1.71])	(5.1 ·10^−1^)	**3.00 [2.17–4.18]**	**4.6 ·10** ^−11^	**1.81 [1.32–2.47]**	**2.0 ·10** ^−4^
rs2187668 *(HLA-DQA1)*	T	6 (32605884)	MN	(0.93 [0.70–1.24])	(6.3 ·10^−1^)	***4.48 [3.32–6.11]***	***2.4 ·10*** ^*−22*^	**2.36 [1.63–3.42]**	**5.9 ·10** ^−6^	(1.89 [1.27–2.80])	(1.6 ·10^−3^)	(0.75 [0.47–1.20])	(2.2 ·10^−1^)
rs1129740 *(HLA-DQA1)*	A	6 (32609105)	SSNS	(1.04 [0.88–1.24])	(6.4 ·10^−1^)	(1.69 [1.30–2.19])	8.4 ·10^−5^	(1.21 [0.89–1.62])	(2.2 ·10^−1^)	2.13 [1.52–2.97]	1.1 ·10^−5^	(1.18 [0.91–1.54])	(2.2 ·10^−1^)
rs9275596 *(HLA-DQB1)*	T	6 (32681631)	IgA	**(1.20 [1.00–1.45])**	*(5.3 ·10* ^*−2*^ *)*	0.52 [0.41–0.67]	3.3 ·10^−7^	(0.57 [0.41–0.77])	(3.4 ·10^−4^)	(1.20 [0.87–1.66])	(2.6 ·10^−1^)	(1.25 [0.94–1.67])	(1.2 ·10^−1^)
rs9277554 *(HLA-DPB1)*	T	6 (33055538)	GPA	(0.75 [0.61–0.92])	(6.5 ·10^−3^)	(1.04 [0.79–1.37])	(7.8 ·10^−1^)	(1.24 [0.91–1.69])	(1.7 ·10^−1^)	(1.05 [0.75–1.46])	(7.9 ·10^−1^)	*0.14 [0.08–0.25]*	*1.7 ·10* ^*−11*^

MN: Membranous nephropathy, IgA: IgA nephropathy, SLE: Systemic lupus erythematosus, GPA: Granulomatosis with polyangiitis, T1DM: Type 1 diabetes mellitus, OR: Odds ratio, CI: Confidence interval. Bold: significant and independent association, italic: previously known association, simple style: significant, but dependent association. Numbers in brackets (): non-significant. For independence tests see Table 3. Significance threshold was set at 2.6 × 10^−4^ (Bonferroni correction: α = 0.05/(38*5)).

Interestingly, several SNPs previously reported as associated with one specific CKD-etiology were associated with one or more additional etiologies of CKD. While *PLA2R1* and *STAT4* were exclusively associated with the previously reported entities MN and SLE, respectively, *TNXB* and several genes in the HLA region were shared across several CKD etiologies (Table 2, Supplementary Table 3). For instance, the known SLE risk variant rs1150754 at *TNXB* was associated not only with CKD attributed to T1DM (OR = 2.53, p = 2.5 × 10^−7^), but also with MN (OR = 2.77, p = 1.9 × 10^−11^).

In order to investigate whether these newly emerging associations represented new findings or emerged because of co-occurrence with previously reported variants for that CKD etiology on shared haplotypes in the HLA region, conditional analyses were performed and linkage disequilibrium calculations were carried out (Table 3, Supplementary Tables 4 and 5). For MN, there were no new independently associated SNPs in the region beyond the original MN risk SNP rs2187668 at *HLA-DQA1*, suggesting that new associations between HLA risk variants for other CKD etiologies and MN were observed because of linkage disequilibrium with a known MN risk variant. Conversely, the association between CKD attributed to T1DM and rs1150754 was independent of previously reported risk variants for other CKD etiologies in the HLA-region (OR = 2.62, p-conditional = 1.0 × 10^−6^). In addition, the IgA risk locus at *HLA-DRB1* was associated independently with GPA conditioned on the known GPA risk variant in *HLA-DPB1* (p = 2.0 × 10^−4^, Table 3). Furthermore, *TNXB* (previously reported for SLE, p = 2.5 × 10^−7^ in GCKD) and *HLA-DRB1* (IgA risk locus, p = 4.6 × 10^−11^ in GCKD) showed independent associations with CKD attributed to T1DM. These associations remained robust in effect size and direction when adjusting for additional previously reported risk variants for T1DM^22–27^ in the HLA region (Supplementary Table 6). LD calculations were consistent with the conditional analyses, with low LD observed between variants for which the effect was not attenuated in conditional analyses (Supplementary Table 4).

**Table 3:** Conditional analyses for independence of SNP signals.

Main SNP	Additional Covariate SNPs	OR [95% CI]	p-value
MN			
rs2187668	—	4.48 [3.32–6.11]	2.4 × 10^−22^
	rs1150754	5.21 [3.32–8.19]	8.1 × 10^−13^
	rs1129740	4.29 [3.09–5.96]	4.1 × 10^−18^
	rs9275596	4.67 [3.16–6.90]	9.6 × 10^−15^
	rs1150754, rs1129740	4,97 [3.10–7.96]	2.7 × 10^−11^
	rs1150754, rs9275596	5.39 [3.24–8.97]	8.6 × 10^−11^
	rs1129740, rs9275596	4.11 [2.36–7.17]	6.2 × 10^−7^
	rs1150754, rs1129740, rs9275596	4.74 [2.49–9.04]	2.2 × 10^−6^
SLE			
rs2187668	—	2.36 [1.63–3.42]	5.9 × 10^−6^
	rs1150754	2.12 [1.22–3.70]	8.0 × 10^−3^
	rs7763262	1.96 [1.30–2.95]	1.3 × 10^−3^
	rs9275596	1.94 [1.26–3.00]	2.7 × 10^−3^
	rs1150754, rs7763262	1.86 [1.04–3.33]	3.6 × 10^−2^
	rs1150754, rs9275596	1.72 [0.94–1.36]	8.0 × 10^−2^
	rs7763262, rs9275596	1.93 [1.25–2.97]	3.0 × 10^−3^
	rs1150754, rs7763262, rs9275596	1.81 [0.97–3.37]	6.1 × 10^−2^
GPA			
rs660895	—	1.81 [1.32–2.47]	2.0 × 10^−4^
	rs9277554	1.73 [1.26–2.38]	7.3 × 10^–4^
rs9277554	—	0.14 [0.08–0.25]	1.7 × 10^−11^
	rs660895	0.14 [0.08–0.25]	2.6 × 10^−11^
T1DM			
rs1150754	—	2.53 [1.78–3.60]	2.5 × 10^−7^
	rs660895	2.75 [1.91–3.98]	7.2 × 10^−8^
	rs1129740	2.12 [1.47–3.05]	5.5 × 10^−5^
	rs660895, rs1129740	2.62 [1.78–3.85]	1.0 × 10^−6^
rs660895	—	3.00 [2.17–4.18]	4.6 × 10^−11^
	rs1150754	3.19 [2.28–4.46]	1.5 × 10^−11^
	rs1129740	2.47 [1.72–3.56]	1.2 × 10^−6^
	rs1150754, rs1129740	2.93 [2.00–4.30]	3.6 × 10^−8^

All SNPs with independent associations with the examined etiology are displayed. Associations were defined as independent if they stayed statistically significant in all performed conditional analyses and ORs did not vary by >20%. Although rs2187668 did not show independence for significant association with SLE throughout, it is shown here because it had the lowest p-value of all SNPs associated with SLE and an OR variation of <20%. For complete data see Supplementary Table 7. MN: Membranous nephropathy, IgA: IgA nephropathy, SLE: Systemic lupus erythematosus, GPA: Granulomatosis with polyangiitis, T1DM: Type 1 diabetes mellitus, OR: Odds ratio, CI: Confidence interval.

Next, two different categories of advanced CKD were examined for 55 SNPs: the first was defined as eGFR < 45 ml/min/1.73 m² (n = 2,245, CKD stage G3b) and the second as UACR ≥ 300 mg/g (n = 1,385, CKD stage A3). These cases were compared to a GCKD control group of 2,245 patients with eGFR > 60 ml/min/1.73 m² and 2,117 patients with UACR < 30 mg/g, respectively. Of the risk loci discovered in the general population, *UMOD* showed significant association (OR = 0.76 per T allele, p < 4.2 × 10^−4^) with CKD stage 3b (Supplementary Table 7) compared to the GCKD control group, after correction for multiple testing. The effect direction was consistent with observations from population-based studies, with the minor T allele associated with better eGFR and lower CKD risk.

In a second set of analyses, the association between the 55 population-based risk loci and CKD for which the treating nephrologists had determined diabetes (n = 653) or hypertension (n = 1086) as the leading cause were examined because they represent the majority of CKD cases in population-based studies. No significant association was detected for both hypertensive nephropathy and diabetic kidney disease (Supplementary Table 8).

## Discussion

This study found genetic associations shared across specific etiologies of CKD. Several known CKD etiology-specific risk loci were replicated in the GCKD study, and risk variants at *TNXB*, *HLA-DQA1*, -*DQB1* and -*DRB1* were independently associated with additional CKD etiologies beyond the ones initially reported.

Based on post-hoc power calculations, our study had excellent power (>99%) to detect some true-positive associations such as the ones between MN nephropathy and the validated risk alleles at *HLA-DQA1* and *PLA2R1*, as well as for some of the new and independent associations with additional disease entities such as the IgA variant rs660895 and T1DM nephropathy (99%) or the MN risk variant rs2187668 and an association with SLE nephropathy (90%). Nevertheless, power was moderate for other combinations, such as the association between the IgA risk variant rs660895 and GPA (50%). *A priori* power calculations were complicated by the fact that it is unclear whether the effect size of a genetic risk variant is the same or similar for different CKD etiologies. Power calculations were therefore provided across a range of case numbers, allele frequencies and effect sizes (Supplementary Table 9) to assess minimum detectable effect sizes. For example, for a case group of 100 patients, there was >80% power to detect an association signal for a SNP with a frequency of 30% and an OR of 2.0. We therefore cannot exclude that there are additional shared genetic risk variants that will only become apparent in future, larger studies that have assembled cases with CKD from different etiologies.

The HLA region is known for containing various risk loci for CKD of different etiologies. Several of them could be examined across CKD etiologies available in the GCKD study, and showed evidence of novel, additional associations that had not been identified at genome-wide significance in genome-wide association studies of these additional etiologies. The associations across CKD etiologies highlight the shared role of the adaptive immune response, and suggest some overlap between etiologies. For example, known risk variants for MN were independently associated with CKD attributed to SLE, which is interesting in light of the histopathological appearance of membranous nephropathy in lupus nephritis class V.

The shared genetic risk variant for CKD attributed to SLE and to T1DM is supported by a report that examined the co-existence of auto-immune diseases^28^. In this report, SLE and T1DM co-exist more often than expected based on the prevalence of both diseases. Several case reports describe different overlapping auto-immune diseases that affect the kidney, such as MN and IgA nephropathy^29^ or MN and further extra-renal autoimmune diseases such as colitis ulcerosa)^30^. More detailed and better powered studies focusing on SNP associations across sub-types of different autoimmune diseases are required to address these important questions in more detail. Furthermore, studies with larger case groups of the respective disease could examine co-incidences of two diseases with overlapping genetic risk factors such as MN/SLE and T1DM/GPA.

The T1DM risk loci found in our study can be interpreted in two ways: either as risk loci for T1DM itself, or as risk loci for diabetic nephropathy resulting from T1DM, or a combination of both. To resolve this question, a control group of T1DM patients without CKD would be required.

We additionally found that the *UMOD* locus known to be associated with kidney function in the general population was also associated with advanced CKD (stage G3b). Experimental evidence links genotype at the *UMOD* risk variant identified in GWAS to altered gene expression and salt-sensitive hypertensive CKD^31^. In support, the risk allele at the *UMOD* variant was significantly associated with stage G3b + CKD in our study, and the association with CKD attributed to hypertension was direction-consistent to the *UMOD* variant reported in previous GWAS of hypertension^32^. This finding illustrates associations across a spectrum of renal function from small changes in eGFR in population-based studies over advanced CKD in the GCKD study to severe renal phenotypes in monogenic diseases caused by loss of function mutations in *UMOD* ^33^.

Strengths of our study include the availability of CKD of different stages and from different etiologies in one study using the same standardized recruitment procedures, and the availability of genome-wide genotype data that allows for carrying out conditional analyses. Other CKD cohorts do not have access to comparable numbers of carefully phenotyped subgroups of patients with as many specific CKD etiologies. Nevertheless, because of limited sample size within subgroups, analyses in our study were restricted to the examination of a predefined number of candidate SNPs and the study of large and moderate genetic effects, rigorously accounting for multiple comparisons. Limitations include the absence of an internal control group of patients without CKD who suffer from the specific diseases that can give risk to CKD, such as T1DM patients without nephropathy.

In conclusion, genetic risk variants for specific etiologies of CKD were shared across some etiologies, suggesting a common mechanism by which the adaptive immune system may contribute to the shared etiologies. In addition, we found the known risk variant at the *UMOD* locus that is associated with CKD in the general population to also associate with advanced stage CKD (G3b+) in the GCKD study, supporting the presence of genetic risk across the spectrum of kidney function.

## Electronic supplementary material

Supplementary Material
