# Supplementary material for: Associations between genetic risk variants for kidney diseases and kidney disease etiology
Source: Sci Rep. 2017 Oct 24;7:13944. doi: 10.1038/s41598-017-13356-6 (PMC5655008; doi:10.1038/s41598-017-13356-6)
Supplement: Supplementary file 1 — Supplementary Material [file 41598_2017_13356_MOESM1_ESM.pdf]

SUPPLEMENTARY MATERIAL

Associations between genetic risk variants for kidney diseases and kidney disease etiology

Sebastian Wunnenburger[1], Ulla T. Schultheiss[1,2], Gerd Walz[2], Birgit Hausknecht[3], Arif B. Ekici[4], Florian Kronenberg[5], Kai-Uwe Eckardt[3], Anna Köttgen[1*], Matthias Wuttke[1*]

Affiliations:
1. Institute of Genetic Epidemiology, Medical Center - University of Freiburg, Faculty of Medicine, Freiburg, Germany
2. Division of Nephrology, University of Freiburg, Faculty of Medicine, Freiburg, Germany
3. Department of Nephrology and Hypertension, University of Erlangen-Nürnberg, Erlangen, Germany
4. Institute of Human Genetics, Friedrich-Alexander-Universität Erlangen-Nürnberg (FAU), Erlangen, Germany
5. Division of Genetic Epidemiology, Department of Medical Genetics, Molecular and Clinical Pharmacology, Medical University of Innsbruck, Innsbruck, Austria

[*]*Indicates joint oversight*

Anna Köttgen, MD, MPH
Institute of Genetic Epidemiology
University Medical Center Freiburg
Hugstetter Straße 49
D-79106 Freiburg
Germany
E-Mail: anna.koettgen@uniklinik-freiburg.de

Contents

Supplementary Table 1. Composition of case and control groups in the GCKD study 3

Supplementary Table 2. Associations of known risk loci for specific CKD etiologies 4

Supplementary Table 3. Associations between CKD etiology associated SNPs and other CKD etiologies 5

Supplementary Table 4: Linkage disequilibrium of selected SNPs in the HLA region in the GCKD cohort 6

Supplementary Table 5. Conditional analyses for independence of SNP signals 7

Supplementary Table 6: Conditional analyses of SNPs associated with CKD from T1DM and previously known T1DM SNPs 9

Supplementary Table 7. Associations between population-based SNPs and advanced CKD (stage G3b+ or A3) 10

Supplementary Table 8. Associations between population-based SNPs and CKD attributed to hypertension and type 2 diabetes mellitus 11

Supplementary Table 9. Power calculations across a range of sample sizes (case numbers), allele frequencies and effect sizes 12

Supplementary Tables

Supplementary Table 1. Composition of case and control groups in the GCKD study

Category	n	n (%) with biopsy
**GCKD case groups**		
CKD stage G3b	2245	NA
CKD stage A3	1385	NA
Nephrosclerosis	1086	84 (7.7)
Type 2 diabetes mellitus	653	28 (4.3)
IgA nephropathy	366	314 (85.8)
Membranous nephropathy	147	140 (95.2)
Systemic lupus erythematosus	128	106 (82.8)
Granulomatosis with polyangiitis	116	81 (69.8)
Type 1 diabetes mellitus	91	4 (4.4)
**GCKD advanced disease control groups**		
CKD stage G1/G2 (eGFR ≥60 ml/min/1.73m²)	1006	NA
CKD stage A1/A2 (UACR <300 mg/g)	2117	NA
**GCKD specific CKD etiology control group**		
Vascular nephropathy (n=1160)		
Renal artery stenosis	49	0 (0.0)
Nephrosclerosis	1086	84 (7.7)
Renal infarct	6	1 (16.7)
Other	19	2 (10.5)
Interstitial nephropathy (n=220)		
Interstitial nephropathy	145	28 (19.3)
Analgesic nephropathy	51	3 (5.9)
Other	24	4 (16.7)
Acute kidney injury (n=62)		
Post ischemic	58	7 (12.1)
Other	4	0 (0.0)
Single kidney (n=133)		
Tumor nephrectomy	62	7 (11.3)
Kidney donor	27	0 (0.0)
Other nephrectomy	31	0 (0.0)
Other	3	0 (0.0)
(Post-)renal diseases (n=90)		
Kidney stones	22	1 (4.5)
Infections	27	1 (3.7)
Neurogenic bladder	3	0 (0.0)
Other	38	2 (5.2)
Sum	1655	140 (8.5)

Supplementary Table 2. Associations of known risk loci for specific CKD etiologies

SNP	Gene	Effect allele	Chromo-some	Position	CKD etiology	OR	[95% CI]		P-value
rs2187668	*HLA-DQA1*	T	6	32,605,884	MN	4.48	3.32	6.11	**2.4E-22**
rs4664308	*PLA2R1*	G	2	160,917,497	MN	0.45	0.34	0.60	**6.7E-08**
rs11150612	*ITGAM-ITGAX*	A	16	31,357,760	IgA	1.14	0.95	1.36	1.6E-01
rs11574637	*ITGAM-ITGAX*	C	16	31,368,874	IgA	0.76	0.59	0.98	3.2E-02
rs12716641	*DEFA*	C	8	6,898,998	IgA	0.89	0.74	1.07	2.0E-01
rs17019602	*VAV3*	G	1	108,188,858	IgA	1.05	0.85	1.30	6.5E-01
rs1794275	*HLA-DQA/B*	A	6	32,671,248	IgA	1.23	0.98	1.54	7.0E-02
rs1883414	*HLA-DPB2*	A	6	33,086,448	IgA	0.88	0.72	1.07	2.0E-01
rs2033562	*KLF10/ODF1*	C	8	103,547,739	IgA	1.15	0.96	1.39	1.4E-01
rs2074038	*ACCS*	T	11	44,087,989	IgA	1.17	0.89	1.54	2.7E-01
rs2412971	*HORMAD2/MTMR3*	A	22	30,494,371	IgA	1.14	0.96	1.35	1.3E-01
rs2523946	*HLA-A*	T	6	29,941,943	IgA	1.15	0.97	1.38	1.1E-01
rs2738048	*DEFA*	G	8	6,822,785	IgA	0.76	0.63	0.92	5.6E-03
rs3115573	*HLA region*	G	6	32,218,843	IgA	1.25	1.05	1.48	1.3E-02
rs3803800	*TNFSF13*	G	17	7,462,969	IgA	0.88	0.72	1.09	2.6E-01
rs4077515	*CARD9*	T	9	139,266,496	IgA	1.15	0.96	1.39	1.2E-01
rs660895	*HLA-DRB1*	G	6	32,577,380	IgA	1.09	0.86	1.37	4.7E-01
rs6677604	*CFHR1,3*	A	1	196,686,918	IgA	0.69	0.54	0.87	1.5E-03
rs7634389	*ST6GAL1*	C	3	186,738,421	IgA	1.30	1.08	1.57	5.9E-03
rs7763262	*HLA-DR–HLA-DQ*	C	6	32,424,882	IgA	1.28	1.05	1.55	1.3E-02
rs9275596	*HLA-DQB1*	T	6	32,681,631	IgA	1.20	1.00	1.45	5.3E-02
rs9314614	*DEFA*	G	8	6,697,731	IgA	1.00	0.84	1.20	9.9E-01
rs9357155	*TAP1/2/PSMB8/9*	A	6	32,809,848	IgA	0.99	0.75	1.32	9.7E-01
rs1129740	*HLA-DQA1*	A	6	32,609,105	SSNS	-	-	-	-
rs10488631	*TNPO3*	C	7	128,594,183	SLE	1.49	0.98	2.27	6.1E-02
rs1150754	*TNXB*	T	6	32,050,758	SLE	1.97	1.37	2.83	2.8E-04
rs4963128	*KIAA1542*	C	11	589,564	SLE	0.94	0.69	1.30	7.3E-01
rs6445975	*PXK*	T	3	58,370,177	SLE	0.94	0.68	1.31	7.3E-01
rs7574865	*STAT4*	G	2	191,964,633	SLE	0.53	0.39	0.73	**9.7E-05**
rs9888739	*ITGAM*	T	16	31,313,253	SLE	1.60	1.10	2.34	1.4E-02
rs12437854	*ESRD*	G	15	94,141,833	T1DM	0.88	0.47	1.68	7.1E-01
rs4972593	*ESRD*	A	2	174,462,854	T1DM	1.29	0.88	1.89	1.9E-01
rs1949829	*COBL*	T	7	51,537,887	GPA	0.75	0.41	1.39	3.6E-01
rs4862110	*DCTD*	C	4	183,751,029	GPA	1.08	0.78	1.49	6.6E-01
rs595018	*CCDC86*	C	11	60,592,276	GPA	1.00	0.72	1.41	9.8E-01
rs7151526	*SERPINA1*	A	14	94,863,636	GPA	1.73	0.99	3.00	5.3E-02
rs7503953	*WSCD1*	C	17	6,141,677	GPA	0.93	0.63	1.40	7.4E-01
rs9277554	*HLA–DPB1*	T	6	33,055,538	GPA	0.14	0.08	0.25	**1.7E-11**

MN: Membranous nephropathy, IgA: IgA nephropathy, SSNS: steroid sensitive nephrotic syndrome, SLE: Systemic lupus erythematosus, GPA: Granulomatosis with polyangiitis, T1DM: Type 1 diabetes mellitus, OR: Odds ratio, CI: Confidence interval. Significance threshold was set at 2.6×10^{-4} (Bonferroni correction: $\alpha < 0.05 / (38*5)$), significant association p-values were marked in bold face.

Supplementary Table 3. Associations between CKD etiology associated SNPs and other CKD etiologies

SNP characteristics				IgA		MN		SLE		GPA		T1DM	
SNP	Gene	Effect allele	Known locus for	OR	p-value	OR	p-value	OR	p-value	OR	p-value	OR	p-value
rs2187668	*HLA-DQA1*	T	MN	0.93	6.3E-01	4.48	**2.4E-22**	2.36	**5.9E-06**	0.75	2.2E-01	1.89	1.6E-03
rs4664308	*PLA2R1*	G	MN	1.04	6.5E-01	0.45	**6.7E-08**	0.83	2.5E-01	1.03	8.1E-01	1.36	4.4E-02
rs11150612	*ITGAM-ITGAX*	A	IgA	1.14	1.6E-01	0.73	2.0E-02	1.03	8.3E-01	1.09	5.4E-01	0.71	4.1E-02
rs11574637	*ITGAM-ITGAX*	C	IgA	0.76	3.2E-02	1.13	4.3E-01	1.56	1.2E-02	0.50	2.6E-03	1.24	2.6E-01
rs12716641	*DEFA*	C	IgA	0.89	2.0E-01	1.06	6.4E-01	0.90	5.1E-01	1.21	1.7E-01	0.75	7.4E-02
rs17019602	*VAV3*	G	IgA	1.05	6.5E-01	1.41	1.7E-02	1.17	3.6E-01	1.42	2.2E-02	0.97	8.7E-01
rs1794275	*HLA-DQA/B*	A	IgA	1.23	7.0E-02	0.61	1.4E-02	0.75	2.0E-01	1.32	9.4E-02	0.64	6.4E-02
rs1883414	*HLA-DPB2*	A	IgA	0.88	2.0E-01	0.74	3.8E-02	1.35	5.1E-02	0.60	1.6E-03	1.04	8.3E-01
rs2033562	*KLF10/ODF1*	C	IgA	1.15	1.4E-01	0.78	5.6E-02	0.92	5.8E-01	0.94	6.8E-01	0.80	1.6E-01
rs2074038	*ACCS*	T	IgA	1.17	2.7E-01	0.94	7.5E-01	1.01	9.8E-01	1.36	1.3E-01	1.04	8.9E-01
rs2412971	*HORMAD2/MTMR3*	A	IgA	1.14	1.3E-01	0.90	3.7E-01	1.00	9.8E-01	1.08	5.7E-01	1.19	2.5E-01
rs2523946	*HLA-A*	T	IgA	1.15	1.1E-01	0.80	9.0E-02	0.72	3.4E-02	0.91	4.7E-01	1.04	7.9E-01
rs2738048	*DEFA*	G	IgA	0.76	5.6E-03	0.98	8.7E-01	1.13	4.1E-01	0.86	3.0E-01	0.98	8.8E-01
rs3115573	*HLA region*	G	IgA	1.25	1.3E-02	0.96	7.4E-01	0.86	3.2E-01	0.82	1.5E-01	1.05	7.3E-01
rs3803800	*TNFSF13*	G	IgA	0.88	2.6E-01	1.01	9.7E-01	1.02	9.3E-01	1.09	6.1E-01	0.99	9.8E-01
rs4077515	*CARD9*	T	IgA	1.15	1.2E-01	0.93	5.7E-01	0.70	2.6E-02	1.02	9.0E-01	1.33	7.3E-02
rs660895	*HLA-DRB1*	G	IgA	1.09	4.7E-01	0.60	1.2E-02	1.15	5.1E-01	1.81	**2.0E-04**	3.00	**4.6E-11**
rs6677604	*CFHR1,3*	A	IgA	0.69	1.5E-03	0.89	4.7E-01	0.99	9.4E-01	1.07	6.7E-01	0.84	3.6E-01
rs7634389	*ST6GAL1*	C	IgA	1.30	5.9E-03	1.06	6.6E-01	1.13	4.4E-01	1.29	7.5E-02	0.94	6.9E-01
rs7763262	*HLA-DR–HLA-DQ*	C	IgA	1.28	1.3E-02	0.67	1.5E-03	0.57	3.0E-04	1.16	3.0E-01	1.21	2.5E-01
rs9275596	*HLA-DQB1*	T	IgA	1.20	5.3E-02	0.52	**3.3E-07**	0.57	3.4E-04	1.25	1.2E-01	1.20	2.6E-01
rs9314614	*DEFA*	G	IgA	1.00	9.9E-01	1.07	5.7E-01	0.91	5.5E-01	0.98	8.6E-01	0.99	9.2E-01
rs9357155	*TAP1/2/PSMB8/9*	A	IgA	0.99	9.7E-01	0.67	7.9E-02	1.18	5.0E-01	1.45	5.2E-02	1.76	5.8E-03
rs1129740	*HLA-DQA1*	A	SSNS	1.04	6.4E-01	1.69	**8.4E-05**	1.21	2.2E-01	1.18	2.2E-01	2.13	**1.1E-05**
rs10488631	*TNPO3*	C	SLE	1.11	4.9E-01	1.14	5.1E-01	1.49	6.1E-02	1.01	9.8E-01	1.09	7.3E-01
rs1150754	*TNXB*	T	SLE	1.07	5.9E-01	2.77	**1.9E-11**	1.97	2.8E-04	0.94	7.7E-01	2.53	**2.5E-07**
rs4963128	*KIAA1542*	C	SLE	1.02	8.2E-01	0.95	7.1E-01	0.94	7.3E-01	0.85	2.7E-01	0.71	2.8E-02
rs6445975	*PXK*	T	SLE	1.06	5.5E-01	1.21	1.9E-01	0.94	7.3E-01	0.89	4.1E-01	0.94	7.2E-01
rs7574865	*STAT4*	G	SLE	0.90	3.4E-01	0.96	7.6E-01	0.53	**9.7E-05**	1.02	9.2E-01	0.85	3.4E-01
rs9888739	*ITGAM*	T	SLE	0.64	4.6E-03	1.06	7.5E-01	1.60	1.4E-02	0.48	7.9E-03	1.18	4.6E-01
rs12437854	*ESRD*	G	T1DM	1.13	4.7E-01	1.21	4.3E-01	1.37	2.7E-01	1.36	2.1E-01	0.88	7.1E-01
rs4972593	*ESRD*	A	T1DM	1.07	6.0E-01	0.88	4.9E-01	1.10	6.5E-01	0.98	9.0E-01	1.29	1.9E-01
rs1949829	*COBL*	T	GPA	1.16	4.0E-01	0.73	2.6E-01	0.98	9.5E-01	0.75	3.6E-01	1.16	6.1E-01
rs4862110	*DCTD*	C	GPA	0.82	8.3E-02	0.92	6.1E-01	0.81	2.8E-01	1.08	6.6E-01	1.13	5.1E-01
rs595018	*CCDC86*	C	GPA	0.97	7.7E-01	0.95	7.2E-01	0.82	2.8E-01	1.00	9.8E-01	0.88	5.1E-01
rs7151526	*SERPINA1*	A	GPA	0.88	5.6E-01	1.98	5.7E-03	0.91	8.1E-01	1.73	5.3E-02	1.43	2.9E-01
rs7503953	*WSCD1*	C	GPA	1.15	3.4E-01	1.05	7.9E-01	0.89	5.9E-01	0.93	7.4E-01	0.66	5.0E-02
rs9277554	*HLA–DPB1*	T	GPA	0.75	6.5E-03	1.04	7.8E-01	1.24	1.7E-01	0.14	**1.7E-11**	1.05	7.9E-01

MN: Membranous nephropathy, IgA: IgA nephropathy, SLE: Systemic lupus erythematosus, GPA: Granulomatosis with polyangiitis, T1DM: Type 1 diabetes mellitus, OR: Odds ratio. Bold: statistically significant association. Significance threshold was set at $2.6x10^{-4}$ (Bonferroni correction: $\alpha < 0.05 / (38*5)$), significant association p-values were marked in bold face.

Supplementary Table 4: Linkage disequilibrium of selected SNPs in the HLA region in the GCKD cohort

		r²							
		rs1150754	rs7763262	rs660895	rs2187668	rs1129740	rs9275596	rs9277554	rs1883414
D'	rs1150754		0.14	<0.01	0.49	0.05	0.12	0.01	<0.01
	rs7763262	0.66		0.10	0.15	0.14	0.61	<0.01	<0.01
	rs660895	0.35	0.95		0.03	0.16	0.11	<0.01	<0.01
	rs2187668	0.74	0.74	1		0.11	0.23	0.02	0.01
	rs1129740	0.64	0.44	1	0.99		0.13	<0.01	<0.01
	rs9275596	0.64	0.82	1	0.97	0.42		0.01	<0.01
	rs9277554	0.16	0.06	0.01	0.2	0.05	0.09		0.15
	rs1883414	0.25	0.05	0.03	0.4	0.01	0.12	0.42	

Linkage disequilibrium of all SNPs on chromosome 6 that were significantly associated with one or more specific CKD etiologies.

Supplementary Table 5. Conditional analyses for independence of SNP signals

SNP	Covariates	OR [95% CI]	p-value	SNP	Covariates	OR [95% CI]	p-value
**MN**				**SLE**			
**rs1150754**	-	2.77 [2.06-3.71]	1.9E-11	**rs1150754**	-	1.97 [1.37-2.83]	2.8E-04
	rs2187668	0.81 [0.51-1.30]	3.9E-01		rs7763262	1.63 [1.10-2.40]	1.4E-02
	rs1129740	2.50 [1.84-3.39]	4.0E-09		rs2187668	1.15 [0.67-1.99]	6.1E-01
	rs9275596	2.28 [1.65-3.14]	6.1E-07		rs9275596	1.64 [1.12-2.42]	1.2E-02
	rs2187668, rs1129740	0.82 [0.51-1.30]	3.9E-01		rs7763262, rs2187668	1.07 [0.61-1.88]	8.1E-01
	rs2187668, rs9275596	0.82 [0.51-1.30]	3.9E-01		rs7763262, rs9275596	1.60 [1.08-2.36]	1.9E-02
	rs1129740, rs9275596	1.41 [0.95-2.10]	8.4E-02		rs2187668, rs9275596	1.17 [0.68-2.02]	5.8E-01
	rs2187668, rs1129740, rs9275596	0.82 [0.51-1.30]	3.9E-01		rs7763262, rs2187668, rs9275596	1.09 [0.61-1.92]	7.8E-01
**rs2187668**	-	4.48 [3.32-6.11]	2.4E-22	**rs7763262**	-	0.57 [0.42-0.77]	3.0E-04
	rs1150754	5.21 [3.32-8.19]	8.1E-13		rs1150754	0.65 [0.47-0.90]	1.0E-02
	rs1129740	4.29 [3.09-5.96]	4.1E-18		rs2187668	0.70 [0.50-0.99]	4.1E-02
	rs9275596	4.67 [3.16-6.90]	9.6E-15		rs9275596	0.72 [0.44-1.18]	1.9E-01
	rs1150754, rs1129740	4,97 [3.10-7.96]	2.7E-11		rs1150754, rs2187668	0.70 [0.50-0.99]	4.6E-02
	rs1150754, rs9275596	5.39 [3.24-8.97]	8.6E-11		rs1150754, rs9275596	0.79 [0.47-1.32]	3.6E-01
	rs1129740, rs9275596	4.11 [2.36-7.17]	6.2E-07		rs2187668, rs9275596	0.73 [0.44-1.21]	2.2E-01
	rs1150754, rs1129740, rs9275596	4.74 [2.49-9.04]	2.2E-06		rs1150754, rs2187668, rs9275596	0.74 [0.44-1.24]	2.6E-01
**rs1129740**	-	1.69 [1.30-2.19]	8.4E-05	**rs2187668**	-	2.36 [1.63-3.42]	5.9E-06
	rs1150754	1.44 [1.10-1.90]	8.2E-03		rs1150754	2.12 [1.22-3.70]	8.0E-03
	rs2187668	1.11 [0.82-1.49]	4.9E-01		rs7763262	1.96 [1.30-2.95]	1.3E-03
	rs9275596	2.57 [1.92-3.45]	2.8E-10		rs9275596	1.94 [1.26-3.00]	2.7E-03
	rs1150754, rs2187668	1.11 [0.82-1.49]	5.0E-01		rs1150754, rs7763262	1.86 [1.04-3.33]	3.6E-02
	rs1150754, rs9275596	2.20 [1.57-3.08]	4.7E-06		rs1150754, rs9275596	1.72 [0.94-1.36]	8.0E-02
	rs2187668, rs9275596	1.14 [0.76-1.70]	5.3E-01		rs7763262, rs9275596	1.93 [1.25-2.97]	3.0E-03
	rs1150754, rs2187668, rs9275596	1.14 [0.76-1.70]	5.3E-01		rs1150754, rs7763262, rs9275596	1.81 [0.97-3.37]	6.1E-02
**rs9275596**	-	0.52 [0.41-0.67]	3.3E-07	**rs9275596**	-	0.57 [0.41-0.77]	3.4E-04
	rs1150754	0.67 [0.51-0.88]	3.7E-03		rs1150754	0.65 [0.46-0.90]	1.0E-02
	rs2187668	1.06 [0.75-1.48]	7.5E-01		rs7763262	0.74 [0.44-1.22]	2.3E-01
	rs1129740	0.36 [0.27-0.47]	6.2E-13		rs2187668	0.74 [0.51-1.06]	1.0E-01
	rs1150754, rs2187668	1.05 [0.75-1.47]	7.7E-01		rs1150754, rs7763262	0.78 [0.46-1.31]	3.4E-01
	rs1150754, rs1129740	0.43 [0.31-0,61]	2.0E-06		rs1150754, rs2187668	0.73 [0.51-1.06]	9.8E-02
	rs2187668, rs1129740	0.96 [0.61-1.51]	8.5E-01		rs7763262, rs2187668	0.95 [0.55-1.62]	8.4E-01
	rs1150754, rs2187668, rs1129740	0.95 [0.60-1.51]	8.4E-01		rs1150754, rs7763262, rs2187668	0.93 [0.54-1.62]	8.0E-01
**T1DM**				**GPA**			
**rs1150754**	-	2.53 [1.78-3.60]	2.5E-07	**rs660895**	-	1.81 [1.32-2.47]	2.0E-04
	rs660895	2.75 [1.91-3.98]	7.2E-08		rs9277554	1.73 [1.26-2.38]	7.3E-04
	rs1129740	2.12 [1.47-3.05]	5.5E-05	**rs9277554**	-	0.14 [0.08-0.25]	1.7E-11
	rs660895, rs1129740	2.62 [1.78-3.85]	1.0E-06		rs660895	0.14 [0.08-0.25]	2.6E-11

**rs660895**	-	3.00 [2.17-4.18]	4.6E-11	
	rs1150754	3.19 [2.28-4.46]	1.5E-11	
	rs1129740	2.47 [1.72-3.56]	1.2E-06	
	rs1150754, rs1129740	2.93 [2.00-4.30]	3.6E-08	
**rs1129740**	-	2.13 [1.52-2.97]	1.1E-05	
	rs1150754	1.85 [1.30-2.62]	5.4E-04	
	rs660895	1.52 [1.04-2.21]	3.0E-02	
	rs1150754, rs660895	1.19 [0.80-1.77]	4.0E-01	

MN: Membranous nephropathy, IgA: IgA nephropathy, SLE: Systemic lupus erythematosus, GPA: Granulomatosis with polyangiitis, T1DM: Type 1 diabetes mellitus, OR: Odds ratio, CI: Confidence interval.

Supplementary Table 6: Conditional analyses of SNPs associated with CKD from T1DM and previously known T1DM SNPs

Conditional analyses rs1150754			Conditional analyses rs660895		
Covariate	OR	p-value	Covariate	OR	p-value
-	2.53	2.5E-07	-	3.01	4.6E-11
rs1015166	2.33	8.3E-06	rs1015166	2.86	4.4E-10
rs11755527	2.53	2.6E-07	rs11755527	3.01	4.7E-11
rs1270942	3.27	3.1E-05	rs1270942	3.36	1.9E-12
rs1980493	2.80	8.0E-06	rs1980493	3.65	6.5E-13
rs2251396	2.10	2.6E-04	rs2251396	2.86	6.0E-10
rs2523989	2.37	3.3E-05	rs2523989	3.15	1.4E-11
rs2647044	2.66	7.7E-05	rs2647044	3.59	6.8E-13
rs2857595	2.60	1.8E-05	rs2857595	3.24	6.2E-12
rs3757247	2.55	2.3E-07	rs3757247	3.01	4.7E-11
rs6931865	2.54	2.5E-07	rs6931865	3.01	4.6E-11
rs886424	2.41	1.7E-04	rs886424	3.22	6.7E-12
rs924043	2.58	1.7E-07	rs924043	3.02	4.1E-11
rs9268645	2.88	1.4E-08	rs9268645	2.91	7.3E-09
rs9272346	2.27	7.4E-06	rs9272346	2.60	8.0E-07
rs9388489	2.54	2.1E-07	rs9388489	3.04	4.0E-11

T1DM: Type 1 diabetes mellitus, OR: Odds ratio

SNPs were selected based on these publications: Hakonarson, Grant et al. 2007 (PMID 17632545); Cooper, Smyth et al. 2008 (PMID 18978792); Barrett, Clayton et al. 2009 (PMID 19430480); Grant, Qu et al. 2009 (PMID 18840781); Bradfield, Qu et al. 2011 (PMID 21980299); Tomer, Dolan et al. 2015 (PMID 25936594))

Supplementary Table 7. Associations between population-based SNPs and advanced CKD (stage G3b+ or A3)

SNP Characteristics			CKD G3b		CKD A3	
SNP	Gene	Effect allele	OR	p-value	OR	p-value
rs10109414	*STC1*	T	1.08	1.8E-01	1.06	3.1E-01
rs10277115	*UNCX*	T	1.08	2.6E-01	1.09	1.6E-01
rs10491967	*TSPAN9*	A	0.99	9.6E-01	1.12	1.8E-01
rs10513801	*ETV5*	G	0.94	4.5E-01	1.08	3.2E-01
rs10774021	*SLC6A13*	T	0.97	6.8E-01	1.03	5.5E-01
rs10794720	*WDR37*	C	0.98	8.3E-01	0.88	1.7E-01
rs10994860	*A1CF*	T	0.99	8.8E-01	0.93	3.0E-01
rs1106766	*INHBC*	T	0.98	7.7E-01	0.99	8.1E-01
rs11078903	*CDK12*	A	1.08	2.4E-01	1.06	3.4E-01
rs11666497	*SIPA1L3*	T	1.08	2.8E-01	0.94	3.2E-01
rs11959928	*DAB2*	A	1.05	4.1E-01	1.03	5.6E-01
rs12124078	*DNAJC16*	G	0.93	2.6E-01	1.08	1.8E-01
rs12136063	*SYPL2*	A	1.01	8.9E-01	0.90	6.0E-02
rs12460876	*SLC7A9*	C	1.00	9.5E-01	0.97	5.2E-01
rs1260326	*GCKR*	C	1.09	1.3E-01	1.04	4.6E-01
rs12917707	*UMOD*	T	0.76	**4.2E-04**	1.04	6.2E-01
rs13538	*ALMS1/NAT8*	G	1.00	9.7E-01	0.95	4.3E-01
rs1394125	*UBE2Q2*	A	1.00	9.6E-01	1.09	1.3E-01
rs163160	*KCNQ1*	G	1.07	3.5E-01	1.08	2.8E-01
rs164748	*DPEP1*	G	1.15	1.7E-02	0.97	5.3E-01
rs17216707	*BCAS1*	C	1.01	9.1E-01	0.93	3.0E-01
rs17319721	*SHROOM3*	A	1.07	2.8E-01	1.17	4.0E-03
rs1801239	*CUBN*	C	1.07	5.0E-01	0.85	5.7E-02
rs2279463	*SLC22A2*	G	1.00	9.8E-01	1.08	3.6E-01
rs228611	*NFKB1*	A	0.99	9.3E-01	0.95	3.4E-01
rs2453580	*SLC47A1*	C	1.14	2.8E-02	0.89	2.9E-02
rs2467853	*SPATA5L1*	G	1.13	3.8E-02	0.91	8.8E-02
rs267734	*ANXA9/LASS2*	C	0.97	6.5E-01	0.92	1.7E-01
rs2712184	*IGFBP5*	A	0.97	6.5E-01	1.00	9.4E-01
rs2802729	*SDCCAG8*	A	1.04	5.3E-01	1.10	6.4E-02
rs2928148	*INO80*	A	0.96	4.6E-01	0.96	4.3E-01
rs347685	*TFDP2*	A	0.97	6.7E-01	0.99	8.5E-01
rs3750082	*KBTBD2*	A	1.07	2.9E-01	1.03	5.5E-01
rs3828890	*MHC region*	G	1.21	6.6E-02	1.00	9.6E-01
rs3850625	*CACNA1S*	A	1.20	5.0E-02	0.88	1.2E-01
rs3925584	*MPPED2*	C	1.01	8.5E-01	0.96	4.2E-01
rs4014195	*AP5B1*	G	1.07	2.9E-01	1.02	6.7E-01
rs4667594	*LRP2*	A	1.03	5.9E-01	1.04	5.2E-01
rs4744712	*PIP5K1B*	C	0.95	3.7E-01	0.95	3.1E-01
rs491567	*WDR72*	C	1.07	3.2E-01	0.96	5.2E-01
rs6088580	*TP53INP2*	C	1.08	1.8E-01	1.07	2.3E-01
rs626277	*DACH1*	C	1.12	6.3E-02	0.95	3.9E-01
rs6420094	*SLC34A1*	G	0.97	6.4E-01	1.04	5.1E-01
rs6431731	*DDX1*	T	0.77	1.3E-01	1.05	7.1E-01
rs6459680	*RNF32*	A	0.91	1.5E-01	1.07	2.4E-01
rs6465825	*TMEM60*	C	0.98	7.2E-01	1.02	6.9E-01
rs6795744	*WNT7A*	A	1.00	9.7E-01	0.94	3.7E-01
rs7422339	*CPS1*	A	1.09	1.6E-01	1.14	2.1E-02
rs7759001	*ZNF204*	A	0.95	4.9E-01	1.08	2.5E-01
rs7805747	*PRKAG2*	A	1.02	7.2E-01	1.07	2.7E-01
rs7956634	*PTPRO*	C	1.01	8.7E-01	1.01	8.9E-01
rs8091180	*NFATC1*	A	0.94	2.9E-01	0.99	8.0E-01
rs881858	*VEGFA*	A	0.94	3.2E-01	1.00	9.8E-01
rs9682041	*SKIL*	T	1.02	8.2E-01	0.99	9.5E-01
rs9895661	*BCAS3*	T	1.01	8.6E-01	0.92	2.2E-01

OR: Odds ratio, CI: Confidence interval. The significance threshold was set at $4.5x10^{-4}$ (Bonferroni correction 0.05/(2 x n(SNPs)), two-sided test). eGFR <45 ml/min/1.73m² cases: n=2245, UACR ≥300 mg/g cases: n=1385, GFR GCKD controls: n=1006 (eGFR >60 ml/min/1.73m²), UACR GCKD controls (UACR <30 mg/g): n=2117.

Supplementary Table 8. Associations between population-based SNPs and CKD attributed to hypertension and type 2 diabetes mellitus

SNP characteristics			Hypertension		Type 2 Diabetes mellitus	
SNP	Gene	Effect allele	OR	p-value	OR	p-value
rs10109414	*STC1*	T	1.02	7.9E-01	1.06	3.7E-01
rs10277115	*UNCX*	T	1.06	5.3E-01	1.04	6.5E-01
rs10491967	*TSPAN9*	A	1.04	7.6E-01	1.08	4.4E-01
rs10513801	*ETV5*	G	0.97	7.6E-01	0.93	4.5E-01
rs10774021	*SLC6A13*	T	0.99	9.0E-01	0.86	3.2E-02
rs10794720	*WDR37*	C	0.95	6.8E-01	0.94	6.0E-01
rs10994860	*A1CF*	T	1.01	9.5E-01	1.01	9.5E-01
rs1106766	*INHBC*	T	0.88	1.8E-01	1.11	1.9E-01
rs11078903	*CDK12*	A	1.00	9.8E-01	0.97	7.0E-01
rs11666497	*SIPA1L3*	T	1.10	3.3E-01	1.09	3.1E-01
rs11959928	*DAB2*	A	1.02	8.2E-01	0.93	2.5E-01
rs12124078	*DNAJC16*	G	0.98	8.4E-01	1.06	4.4E-01
rs12136063	*SYPL2*	A	0.93	3.9E-01	0.96	5.9E-01
rs12460876	*SLC7A9*	C	0.99	8.9E-01	1.00	9.4E-01
rs1260326	*GCKR*	C	1.13	1.1E-01	1.07	3.0E-01
rs12917707	*UMOD*	T	0.90	3.3E-01	1.12	2.1E-01
rs13538	*ALMS1/NAT8*	G	0.89	2.1E-01	1.03	7.2E-01
rs1394125	*UBE2Q2*	A	1.04	5.9E-01	1.04	5.8E-01
rs163160	*KCNQ1*	G	1.02	8.1E-01	0.98	8.0E-01
rs164748	*DPEP1*	G	0.90	1.6E-01	1.08	2.3E-01
rs17216707	*BCAS1*	C	1.01	9.4E-01	0.96	6.2E-01
rs17319721	*SHROOM3*	A	0.92	3.0E-01	0.98	7.6E-01
rs1801239	*CUBN*	C	0.95	6.9E-01	1.26	2.3E-02
rs2279463	*SLC22A2*	G	1.20	1.2E-01	0.87	1.7E-01
rs228611	*NFKB1*	A	1.00	9.7E-01	0.98	7.3E-01
rs2453580	*SLC47A1*	C	1.15	7.3E-02	1.03	7.0E-01
rs2467853	*SPATA5L1*	G	0.85	3.6E-02	0.92	2.1E-01
rs267734	*ANXA9/LASS2*	C	1.04	6.9E-01	1.11	2.1E-01
rs2712184	*IGFBP5*	A	1.04	6.2E-01	1.12	1.0E-01
rs2802729	*SDCCAG8*	A	0.83	1.7E-02	0.91	1.5E-01
rs2928148	*INO80*	A	0.88	8.9E-02	1.18	1.4E-02
rs347685	*TFDP2*	A	1.04	6.5E-01	1.04	6.1E-01
rs3750082	*KBTBD2*	A	0.89	1.3E-01	1.02	8.2E-01
rs3828890	*MHC region*	G	1.05	7.3E-01	0.96	7.5E-01
rs3850625	*CACNA1S*	A	1.00	1.0E+00	1.13	2.2E-01
rs3925584	*MPPED2*	C	1.01	9.5E-01	1.12	9.0E-02
rs4014195	*AP5B1*	G	1.21	1.5E-02	0.93	2.5E-01
rs4667594	*LRP2*	A	1.08	3.2E-01	0.94	3.4E-01
rs4744712	*PIP5K1B*	C	0.94	4.0E-01	1.14	5.5E-02
rs491567	*WDR72*	C	1.01	8.9E-01	0.99	9.3E-01
rs6088580	*TP53INP2*	C	0.88	1.0E-01	1.03	7.1E-01
rs626277	*DACH1*	C	1.00	9.9E-01	1.05	4.8E-01
rs6420094	*SLC34A1*	G	0.97	6.6E-01	0.96	5.8E-01
rs6431731	*DDX1*	T	1.38	1.1E-01	1.42	7.4E-02
rs6459680	*RNF32*	A	1.03	7.3E-01	1.03	6.8E-01
rs6465825	*TMEM60*	C	0.91	2.3E-01	1.04	5.3E-01
rs6795744	*WNT7A*	A	1.18	1.2E-01	0.97	7.9E-01
rs7422339	*CPS1*	A	0.90	2.3E-01	0.99	8.6E-01
rs7759001	*ZNF204*	A	1.04	6.6E-01	1.10	2.2E-01
rs7805747	*PRKAG2*	A	0.92	3.2E-01	0.92	2.6E-01
rs7956634	*PTPRO*	C	1.02	8.4E-01	0.95	5.2E-01
rs8091180	*NFATC1*	A	0.99	9.2E-01	1.01	9.0E-01
rs881858	*VEGFA*	A	0.97	7.4E-01	0.83	1.1E-02
rs9682041	*SKIL*	T	1.01	9.5E-01	1.15	1.8E-01
rs9895661	*BCAS3*	T	0.94	5.1E-01	1.00	9.9E-01

OR: Odds ratio, CI: Confidence interval. The significance threshold was set at 4.5×10^{-4} ($\alpha = 0.05 / (55*2)$, Bonferroni correction, two-sided test). CKD from hypertension (nephrosclerosis) cases: n=1086, CKD from type 2 diabetes mellitus: n=653, GCKD controls: n=569 for hypertension (specific CKD etiology control group excluding nephrosclerosis), and n=1655 for T2DM.

Supplementary Table 9. Power calculations across a range of sample sizes (case numbers), allele frequencies and effect sizes

Freq	OR	N	Power	Freq	OR	N	Power	Freq	OR	N	Power	Freq	OR	N	Power
0.10	1.0	100	0.0%	0.15	1.0	100	0.0%	0.20	1.0	100	0.0%	0.25	1.0	100	0.0%
0.10	1.0	200	0.0%	0.15	1.0	200	0.0%	0.20	1.0	200	0.0%	0.25	1.0	200	0.0%
0.10	1.0	300	0.0%	0.15	1.0	300	0.0%	0.20	1.0	300	0.0%	0.25	1.0	300	0.0%
0.10	1.0	400	0.0%	0.15	1.0	400	0.0%	0.20	1.0	400	0.0%	0.25	1.0	400	0.0%
0.10	1.1	100	0.1%	0.15	1.1	100	0.1%	0.20	1.1	100	0.1%	0.25	1.1	100	0.1%
0.10	1.1	200	0.1%	0.15	1.1	200	0.1%	0.20	1.1	200	0.2%	0.25	1.1	200	0.2%
0.10	1.1	300	0.2%	0.15	1.1	300	0.2%	0.20	1.1	300	0.3%	0.25	1.1	300	0.4%
0.10	1.1	400	0.2%	0.15	1.1	400	0.3%	0.20	1.1	400	0.5%	0.25	1.1	400	0.6%
0.10	1.2	100	0.2%	0.15	1.2	100	0.3%	0.20	1.2	100	0.4%	0.25	1.2	100	0.5%
0.10	1.2	200	0.5%	0.15	1.2	200	0.9%	0.20	1.2	200	1.4%	0.25	1.2	200	1.8%
0.10	1.2	300	1.0%	0.15	1.2	300	1.9%	0.20	1.2	300	3.0%	0.25	1.2	300	4.0%
0.10	1.2	400	1.8%	0.15	1.2	400	3.4%	0.20	1.2	400	5.3%	0.25	1.2	400	7.2%
0.10	1.3	100	0.6%	0.15	1.3	100	1.0%	0.20	1.3	100	1.5%	0.25	1.3	100	2.0%
0.10	1.3	200	2.0%	0.15	1.3	200	3.9%	0.20	1.3	200	6.0%	0.25	1.3	200	8.1%
0.10	1.3	300	4.6%	0.15	1.3	300	9.1%	0.20	1.3	300	14.0%	0.25	1.3	300	18.6%
0.10	1.3	400	8.3%	0.15	1.3	400	16.4%	0.20	1.3	400	24.8%	0.25	1.3	400	32.1%
0.10	1.4	100	1.5%	0.15	1.4	100	2.8%	0.20	1.4	100	4.2%	0.25	1.4	100	5.6%
0.10	1.4	200	6.0%	0.15	1.4	200	11.7%	0.20	1.4	200	17.7%	0.25	1.4	200	23.1%
0.10	1.4	300	13.9%	0.15	1.4	300	26.2%	0.20	1.4	300	37.7%	0.25	1.4	300	46.8%
0.10	1.4	400	24.5%	0.15	1.4	400	43.3%	0.20	1.4	400	58.0%	0.25	1.4	400	68.2%
0.10	1.5	100	3.3%	0.15	1.5	100	6.4%	0.20	1.5	100	9.6%	0.25	1.5	100	12.6%
0.10	1.5	200	13.9%	0.15	1.5	200	26.0%	0.20	1.5	200	37.0%	0.25	1.5	200	45.7%
0.10	1.5	300	30.5%	0.15	1.5	300	51.3%	0.20	1.5	300	66.1%	0.25	1.5	300	75.4%
0.10	1.5	400	49.0%	0.15	1.5	400	72.6%	0.20	1.5	400	85.2%	0.25	1.5	400	91.3%
0.10	2.0	100	36.6%	0.15	2.0	100	57.0%	0.20	2.0	100	70.0%	0.25	2.0	100	77.5%
0.10	2.0	200	84.7%	0.15	2.0	200	96.1%	0.20	2.0	200	98.8%	0.25	2.0	200	99.5%
0.10	2.0	300	98.1%	0.15	2.0	300	99.9%	0.20	2.0	300	100.0%	0.25	2.0	300	100.0%
0.10	2.0	400	99.9%	0.15	2.0	400	100.0%	0.20	2.0	400	100.0%	0.25	2.0	400	100.0%
0.10	2.5	100	82.3%	0.15	2.5	100	94.4%	0.20	2.5	100	97.7%	0.25	2.5	100	98.8%
0.10	2.5	200	99.8%	0.15	2.5	200	100.0%	0.20	2.5	200	100.0%	0.25	2.5	200	100.0%
0.10	2.5	300	100.0%	0.15	2.5	300	100.0%	0.20	2.5	300	100.0%	0.25	2.5	300	100.0%
0.10	2.5	400	100.0%	0.15	2.5	400	100.0%	0.20	2.5	400	100.0%	0.25	2.5	400	100.0%
0.10	3.0	100	97.9%	0.15	3.0	100	99.7%	0.20	3.0	100	99.9%	0.25	3.0	100	100.0%
0.10	3.0	200	100.0%	0.15	3.0	200	100.0%	0.20	3.0	200	100.0%	0.25	3.0	200	100.0%
0.10	3.0	300	100.0%	0.15	3.0	300	100.0%	0.20	3.0	300	100.0%	0.25	3.0	300	100.0%
0.10	3.0	400	100.0%	0.15	3.0	400	100.0%	0.20	3.0	400	100.0%	0.25	3.0	400	100.0%
0.10	3.5	100	99.9%	0.15	3.5	100	100.0%	0.20	3.5	100	100.0%	0.25	3.5	100	100.0%
0.10	3.5	200	100.0%	0.15	3.5	200	100.0%	0.20	3.5	200	100.0%	0.25	3.5	200	100.0%
0.10	3.5	300	100.0%	0.15	3.5	300	100.0%	0.20	3.5	300	100.0%	0.25	3.5	300	100.0%
0.10	3.5	400	100.0%	0.15	3.5	400	100.0%	0.20	3.5	400	100.0%	0.25	3.5	400	100.0%
0.10	4.0	100	100.0%	0.15	4.0	100	100.0%	0.20	4.0	100	100.0%	0.25	4.0	100	100.0%
0.10	4.0	200	100.0%	0.15	4.0	200	100.0%	0.20	4.0	200	100.0%	0.25	4.0	200	100.0%
0.10	4.0	300	100.0%	0.15	4.0	300	100.0%	0.20	4.0	300	100.0%	0.25	4.0	300	100.0%
0.10	4.0	400	100.0%	0.15	4.0	400	100.0%	0.20	4.0	400	100.0%	0.25	4.0	400	100.0%
0.10	4.5	100	100.0%	0.15	4.5	100	100.0%	0.20	4.5	100	100.0%	0.25	4.5	100	100.0%
0.10	4.5	200	100.0%	0.15	4.5	200	100.0%	0.20	4.5	200	100.0%	0.25	4.5	200	100.0%
0.10	4.5	300	100.0%	0.15	4.5	300	100.0%	0.20	4.5	300	100.0%	0.25	4.5	300	100.0%
0.10	4.5	400	100.0%	0.15	4.5	400	100.0%	0.20	4.5	400	100.0%	0.25	4.5	400	100.0%
0.10	5.0	100	100.0%	0.15	5.0	100	100.0%	0.20	5.0	100	100.0%	0.25	5.0	100	100.0%
0.10	5.0	200	100.0%	0.15	5.0	200	100.0%	0.20	5.0	200	100.0%	0.25	5.0	200	100.0%
0.10	5.0	300	100.0%	0.15	5.0	300	100.0%	0.20	5.0	300	100.0%	0.25	5.0	300	100.0%
0.10	5.0	400	100.0%	0.15	5.0	400	100.0%	0.20	5.0	400	100.0%	0.25	5.0	400	100.0%

Freq	OR	N	Power	Freq	OR	N	Power	Freq	OR	N	Power
0.30	1.0	100	0.0%	0.35	1.0	100	0.0%	0.40	1.0	100	0.0%
0.30	1.0	200	0.0%	0.35	1.0	200	0.0%	0.40	1.0	200	0.0%
0.30	1.0	300	0.0%	0.35	1.0	300	0.0%	0.40	1.0	300	0.0%
0.30	1.0	400	0.0%	0.35	1.0	400	0.0%	0.40	1.0	400	0.0%
0.30	1.1	100	0.1%	0.35	1.1	100	0.1%	0.40	1.1	100	0.1%
0.30	1.1	200	0.3%	0.35	1.1	200	0.3%	0.40	1.1	200	0.3%
0.30	1.1	300	0.4%	0.35	1.1	300	0.5%	0.40	1.1	300	0.5%
0.30	1.1	400	0.7%	0.35	1.1	400	0.8%	0.40	1.1	400	0.9%
0.30	1.2	100	0.6%	0.35	1.2	100	0.7%	0.40	1.2	100	0.8%
0.30	1.2	200	2.2%	0.35	1.2	200	2.5%	0.40	1.2	200	2.7%
0.30	1.2	300	4.9%	0.35	1.2	300	5.6%	0.40	1.2	300	6.1%
0.30	1.2	400	8.9%	0.35	1.2	400	10.3%	0.40	1.2	400	11.2%
0.30	1.3	100	2.4%	0.35	1.3	100	2.7%	0.40	1.3	100	2.9%
0.30	1.3	200	9.9%	0.35	1.3	200	11.3%	0.40	1.3	200	12.1%
0.30	1.3	300	22.5%	0.35	1.3	300	25.3%	0.40	1.3	300	27.1%
0.30	1.3	400	37.8%	0.35	1.3	400	41.9%	0.40	1.3	400	44.4%
0.30	1.4	100	6.8%	0.35	1.4	100	7.6%	0.40	1.4	100	8.2%
0.30	1.4	200	27.4%	0.35	1.4	200	30.5%	0.40	1.4	200	32.2%
0.30	1.4	300	53.4%	0.35	1.4	300	57.7%	0.40	1.4	300	60.1%
0.30	1.4	400	74.6%	0.35	1.4	400	78.5%	0.40	1.4	400	80.5%
0.30	1.5	100	15.0%	0.35	1.5	100	16.7%	0.40	1.5	100	17.6%
0.30	1.5	200	51.9%	0.35	1.5	200	55.8%	0.40	1.5	200	57.8%
0.30	1.5	300	81.0%	0.35	1.5	300	84.1%	0.40	1.5	300	85.6%
0.30	1.5	400	94.3%	0.35	1.5	400	95.7%	0.40	1.5	400	96.3%
0.30	2.0	100	81.5%	0.35	2.0	100	83.4%	0.40	2.0	100	83.9%
0.30	2.0	200	99.7%	0.35	2.0	200	99.8%	0.40	2.0	200	99.8%
0.30	2.0	300	100.0%	0.35	2.0	300	100.0%	0.40	2.0	300	100.0%
0.30	2.0	400	100.0%	0.35	2.0	400	100.0%	0.40	2.0	400	100.0%
0.30	2.5	100	99.2%	0.35	2.5	100	99.3%	0.40	2.5	100	99.2%
0.30	2.5	200	100.0%	0.35	2.5	200	100.0%	0.40	2.5	200	100.0%
0.30	2.5	300	100.0%	0.35	2.5	300	100.0%	0.40	2.5	300	100.0%
0.30	2.5	400	100.0%	0.35	2.5	400	100.0%	0.40	2.5	400	100.0%
0.30	3.0	100	100.0%	0.35	3.0	100	100.0%	0.40	3.0	100	100.0%
0.30	3.0	200	100.0%	0.35	3.0	200	100.0%	0.40	3.0	200	100.0%
0.30	3.0	300	100.0%	0.35	3.0	300	100.0%	0.40	3.0	300	100.0%
0.30	3.0	400	100.0%	0.35	3.0	400	100.0%	0.40	3.0	400	100.0%
0.30	3.5	100	100.0%	0.35	3.5	100	100.0%	0.40	3.5	100	100.0%
0.30	3.5	200	100.0%	0.35	3.5	200	100.0%	0.40	3.5	200	100.0%
0.30	3.5	300	100.0%	0.35	3.5	300	100.0%	0.40	3.5	300	100.0%
0.30	3.5	400	100.0%	0.35	3.5	400	100.0%	0.40	3.5	400	100.0%
0.30	4.0	100	100.0%	0.35	4.0	100	100.0%	0.40	4.0	100	100.0%
0.30	4.0	200	100.0%	0.35	4.0	200	100.0%	0.40	4.0	200	100.0%
0.30	4.0	300	100.0%	0.35	4.0	300	100.0%	0.40	4.0	300	100.0%
0.30	4.0	400	100.0%	0.35	4.0	400	100.0%	0.40	4.0	400	100.0%
0.30	4.5	100	100.0%	0.35	4.5	100	100.0%	0.40	4.5	100	100.0%
0.30	4.5	200	100.0%	0.35	4.5	200	100.0%	0.40	4.5	200	100.0%
0.30	4.5	300	100.0%	0.35	4.5	300	100.0%	0.40	4.5	300	100.0%
0.30	4.5	400	100.0%	0.35	4.5	400	100.0%	0.40	4.5	400	100.0%
0.30	5.0	100	100.0%	0.35	5.0	100	100.0%	0.40	5.0	100	100.0%
0.30	5.0	200	100.0%	0.35	5.0	200	100.0%	0.40	5.0	200	100.0%
0.30	5.0	300	100.0%	0.35	5.0	300	100.0%	0.40	5.0	300	100.0%
0.30	5.0	400	100.0%	0.35	5.0	400	100.0%	0.40	5.0	400	100.0%

$\alpha = 0.000263$, $K_P = 0.0001$, n(controls): 1,655
